# Towards High Accuracy Pedestrian Detection on Edge GPUs

**DOI:** 10.3390/s22165980

**Published:** 2022-08-10

**Authors:** Huaping Zhou, Tao Wu, Kelei Sun, Chunjiong Zhang

**Affiliations:** 1 School of Computer Science and Engineering, Anhui University of Science and Technology, Huainan 232001, China; 2 College of Electronics and Information Engineering, Tongji University, Shanghai 200092, China

**Keywords:** YOLOv4-tiny, attention mechanism, feature fusion, lightweight

## Abstract

Despite the rapid development of pedestrian detection algorithms, the balance between detection accuracy and efficiency is still far from being achieved due to edge GPUs (low computing power) limiting the parameters of the model. To address this issue, we propose the YOLOv4-TP-Tiny based on the YOLOv4 model, which mainly includes two modules, two-dimensional attention (TA) and pedestrian-based feature extraction (PFM). First, we integrate the TA mechanism into the backbone network, which increases the attention of the network to the visible area of pedestrians and improves the accuracy of pedestrian detection. Then, the PFM is used to replace the original spatial pyramid pooling (SPP) structure in the YOLOv4 to obtain the YOLOv4-TP algorithm, which can adapt to different sizes of people to obtain higher detection accuracy. To maintain detection speed, we replaced the normal convolution with a ghost network with a TA mechanism, resulting in more feature maps with fewer parameters. We constructed a one-way multi-scale feature fusion structure to replace the down-sampling process, thereby reducing network parameters to obtain the YOLOv4-TP-Tiny model. The experimental results show that the YOLOv4-TP-tiny has 58.3% AP and 31 FPS in the winder person pedestrian dataset. With the same hardware conditions and dataset, the AP of the YOLOv4-tiny is 55.9%, and the FPS is 29.

## 1. Introduction

As a focus of object detection research, pedestrian detection research [1] is becoming increasingly popular due to advances in deep learning. The goal of pedestrian detection is to use deep learning models to analyze the collected picture or video data to determine the location information of pedestrian targets. To build a safer, smarter and more harmonious city, many places need to analyze the behavior of pedestrians. Therefore, pedestrian detection technology has application prospects in video surveillance [2], safe driving [3], outdoor security [4] and other fields.

Due to the rapid development of deep learning in recent years, many deep-learning-based object detection methods have achieved exciting results. The research of real-time object detection is mainly a single-stage network model, which is located and classified in the same network to obtain the final detection results, such as the YOLO, SSD [5] and Mobilenet [6] networks. However, due to the high computational requirements of these networks, their detection speeds are not ideal on edge GPUs. Many studies have designed more lightweight networks. The YOLO-Ret [7] uses the detection head of the YOLOv4 and the backbone network of Mobilenet for lightweighting. Luis et al. [8] proposed a combination of SSD and Mobilenet methods for lightweighting, and they have achieved good results, but in a complex pedestrian environment, the performance is not very good.

Therefore, we propose the YOLOv4-TP-Tiny model to achieve target detection in complex pedestrian environments. We also use the YOLOv4 as the detection head, while using a lightweight network for real-time object detection. The YOLOv4 [9] model’s detection speed and accuracy have reached state of the art, but it also failed to achieve high-precision pedestrian detection in real time on the edge GPUs. To achieve real-time target detection, Jiang proposed that the YOLOv4-Tiny [10] achieved good results. Since it is not a network designed for pedestrian detection, it does not perform very well in complex pedestrian environments. The YOLOv4-TP-Tiny is designed for pedestrian detection, which can detect pedestrians of different sizes and at the same time perform well in pedestrians with heavy obstruction. Most importantly, it can be detected in real time at edge GPUs.

The YOLOv4-TP-Tiny aims to improve the extraction of pedestrian features and the fusion of these two aspects and to improve the detection accuracy as much as possible while ensuring real-time. Our contributions include:

- We propose that the TA module improves pedestrian feature extraction, which utilizes the idea of dynamically adjusting k neighborhoods to improve the extraction ability of pedestrian features in the convolutional block attention module (CBAM) [11]. Then we integrate TA into the backbone network to increase the network’s attention to the pedestrian visible area.
- According to the a priori pedestrian aspect ratio information, different sizes of cavity convolution are designed to replace the pooling layer in SPP. PFM is constructed from the width of the network, which enhances the ability of the network to extract multi-scale features of pedestrians and thus improve the detection accuracy.
- To keep detection speed, the down-sampling process in PAN [12] is abandoned during feature fusion. The ghost-module-integrating the TA mechanism is used to build a one-way multi-scale feature fusion structure to realize the lightweight of the model.

## 2. Related Work

Modeling in object detection has been dominated by convolutional neural networks (CNN), and many popular pedestrian detection algorithms are implemented by a CNN. There are two main ideas for improving detection effect.

### 2.1. Optimization of Network Structure

Lei et al. [13] proposed a Multi-Stream Region Proposal Network based on pedestrian area information. The model extracts different features according to the unobstructed area of the human body. First, the model integrates the pedestrian’s visible area feature map through a fusion network model, and then it sets the Region Proposal Network (RPN) to obtain the region proposals in the feature map. Liu et al. [14] proposed the Cross-Stage-Partial-connections (CSP) algorithm, which is based on the prediction of pedestrian center and scale. Instead of using a sliding window for detection, it can predict the pedestrian center coordinates and scale through a full convolution structure. Based on the Faster R-CNN algorithm, Zhang et al. [15] reused the cascaded integrated learning boosted forest algorithm to classify the candidate pedestrian frames generated by RPN, which reduces the false detection rate of small pedestrians. Li et al. [16] combined a large network and a small subnetwork into a unified architecture to handle pedestrians of various sizes in the image. Yan et al. [17] proposed different networks to train pedestrians at different scales. In the final detection stage, the results in the two networks are incorporated with each other to improve the detection accuracy. While the above study improves the accuracy of pedestrian detection, it cannot be detected in real time on edge GPUs.

### 2.2. Model Lightweight

There is also a lot of research on lightweight models. The earliest lightweight neural network is SqueezeNet [18] proposed in 2016. It mainly uses more 1 × 1 convolution to replace 3 × 3 convolution in the network structure, reducing the number of channels before feature extraction and improving the network detection accuracy by delaying down-sampling. MobileNet adopted the idea of depth-wise separable convolution as the guidance and used the alternation of deep convolution and pointwise convolution to replace the original convolution without losing too much accuracy and reducing the amount of model calculation, which is applied to embedded devices such as mobile phones. MobileNetV2 [19] adopted residuals to expand the number of features based on MobileNetV1 to prevent the ReLU activation function from damaging features through linear bottlenecks. Xception [20] improved the PerceptionV3 network by replacing the standard convolution operation of the network with pointwise convolution and then deep convolution. It also adds a ReLU activation function between the two convolution layers. Group pointwise convolution and channel shuffle are introduced into the network structure of ShuffleNetV1 [21], which reduces the amount of calculation and has high efficiency in mobile terminals such as mobile phones. ShuffleNetV2 [22] put forward four practical guidelines for designing an efficient CNN network, and improved ShuffleNetV1 based on this guideline to ensure high precision and high speed. GhostNet [23] discovered correlations between different channels of the feature layer and copied the channels of the feature layer through simple linear operations, generating redundant feature maps, which greatly reduced the amount of computation. Although the above study obtained very small network parameters, the detection accuracy is not high. The YOLOv4-TP-Tiny proposed in this paper takes into account the idea of the lightweight model so that the model can improve the detection accuracy as much as possible while meeting the real-time requirements.

## 3. Method

### 3.1. Overall Architecture

**Figure 1:**
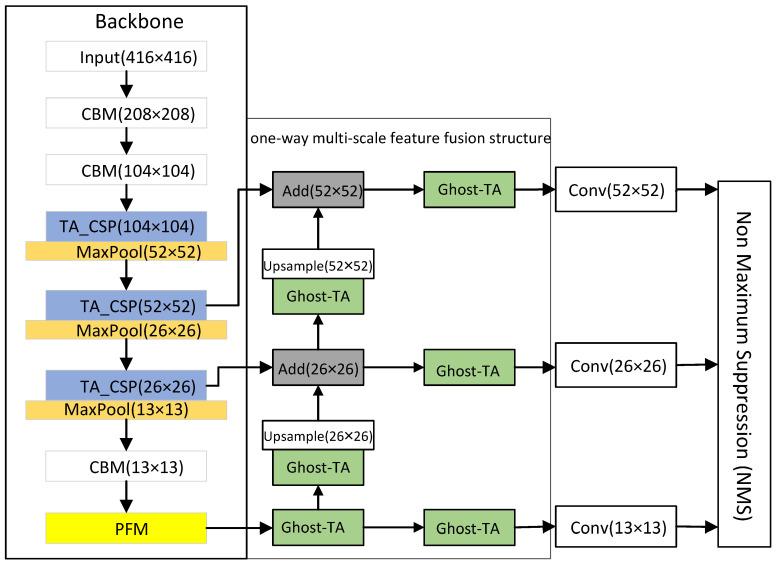
The YOLOv4-TP-tiny network structure.

The YOLOv4-TP-Tiny presented in this article consists of a backbone network, a neck network and an output header. This is shown in Figure 1. The input to the network is the picture that needs to be detected, and then the feature is extracted through our improved backbone network feature extraction. The resulting feature map pays more attention to the area of pedestrians and can accommodate pedestrians of different sizes.

In the neck network, the ideas from Ghost-Net are adopted, and TA is introduced to achieve better feature fusion with the previously obtained multi-scale image features. Non maximum suppression (NMS) is used in the final output head to obtain the final detection frame to complete the task of pedestrian detection. These are described in detail in the next two subsections; they are mainly divided into the improvement of detection accuracy and speed.

### 3.2. Precision Optimization

**Figure 2:**
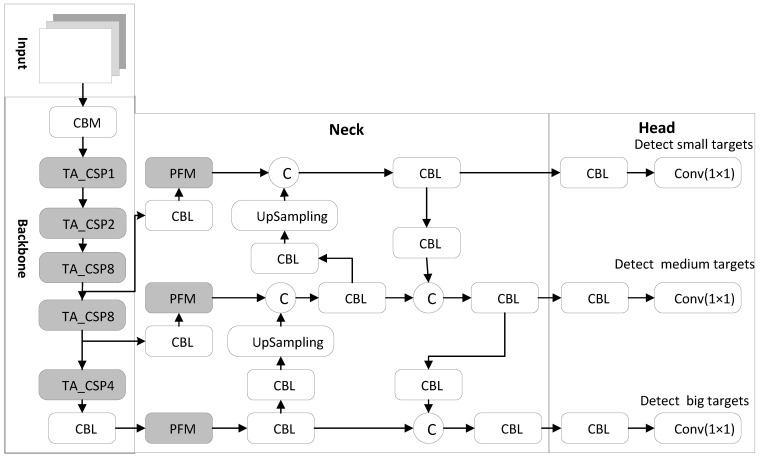
Overall network structure of the YOLOv4-TP.

The pedestrian detection algorithm proposed in this paper is called the YOLOv4-TP, where T and P refer to the proposed TA module and PFM module, respectively. The structure of the YOLOv4-TP model is shown in Figure 1, with the gray part being an improved module compared to the YOLOv4. In Figure 2, CBM and CBL are the smallest convolutional module structures, consisting of a convolutional layer (conv), a batch normalization layer (BN) and an activation function layer. The difference between them is the activation function, the Mish function and the Leaky_ReLU function, respectively. In the figure, © refers to the splicing mode between feature maps being channel concatenate. During the convolution process, the CSPDarkNet53 backbone network of the YOLOv4 algorithm down-samples the input image, gradually reducing the size of the feature image but losing some feature information. Moreover, the backbone network pays the same attention to the visible area of dense pedestrians as other areas, resulting in incomplete pedestrian information extracted. Its performance is not ideal when it is directly applied to the pedestrian detection in dense scenes. Therefore, we integrated the TA mechanism designed in this paper into the CSPDarkNet53 backbone network to improve the network’s attention to the pedestrian area.

#### 3.2.1. CSPDarkNet53 Network

**Figure 3:**
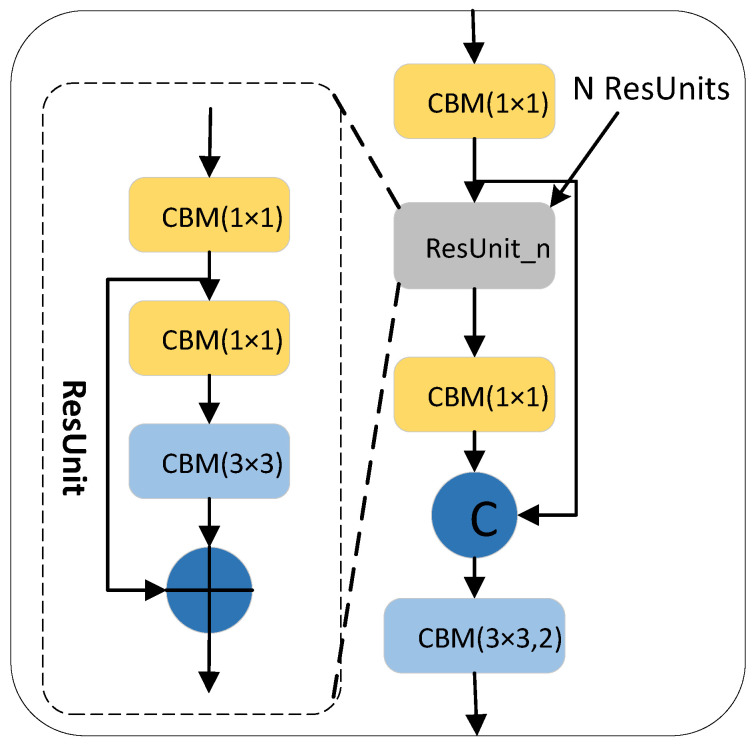
The structure of CSP_n.

The CSPDarkNet53 backbone network is a 53-layer convolution network composed of an ordinary CBM convolution module and multiple cross-stage partial (CSP) [24] networks structures. Among them, the CSP structure adds a residual edge based on the connection of multiple residual units (ResUnit) [25]. Without too much processing, the residual edge is directly connected with the original ResUnit for channel concatenate. To ensure that the output feature map of the residual unit can be channel concatenated with the residual edge, a 1 × 1 CBM convolution block is used in the CSP structure to adjust the channel of the output feature map in the residual unit, which is consistent with the channel of the residual edge. To reduce the computation, inside each ResUnit residual unit, the 1 × 1 CBM convolutional layer is used to reduce the channel in the input feature map and then add it to the input feature map element-wise after being processed by the 3 × 3 CBM convolution layer. The convolution kernel size in the last CBM convolution module on the CSP structure is 3 × 3, and the stride is 2, which is used to scale the resolution in the feature layer. The CSP structure composed of n residual units is called the CSP_n structure, as shown in Figure 3.

The process of feature extraction with the backbone network composed by the CSP structure is shown in Formulas (1) and (2):

(1) $$y_{m}=f_{m}\left(y_{m}-1\right)=f_{m}\left(f_{m}-1\left(\cdots f_{1}\left(x\right)\right)\right),$$

(2) $$F=\{y_{1},y_{2},\cdots y_{m}\}.$$

In the formulas, *y_m_* represents the output feature map of the mth CSP structure, *F* represents a collection of output feature maps in the network, *x* represents the network input, and *f*${}_{m}\left(\cdot \right)$ represents the network structure with m CSP_n structures.

In the CSPDarknet53 backbone network in the YOLOv4-TP algorithm, 5 CSP structures are used, where each CSP structure contains the number of residual units n 1, 2, 8, 8 and 4, respectively.

#### 3.2.2. TA Module

**Figure 4:**
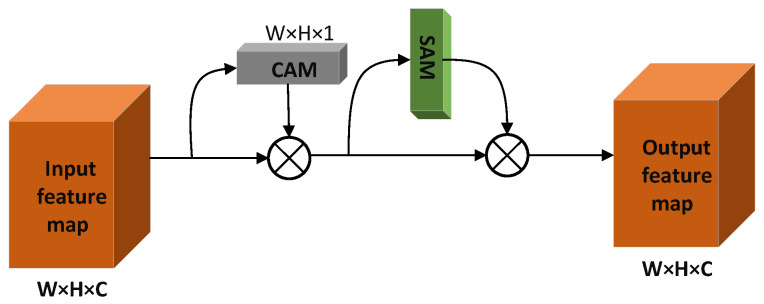
The structure of the TA.

Drawing on the idea of Efficient Channel Attention (ECA) [26], the channel attention block in the CBAM is improved to build the TA mechanism. The TA mechanism is structurally the same as the CBAM, with both using channel and spatial dimensions to enhance feature map weights. The TA mechanism first uses the input feature map to obtain the weight vector of the CAM (Channel Attention mechanism) from the channel dimension and then strengthens the channel weight in the original input feature map. It obtains the weight map of the SAM from the spatial dimension and strengthens the spatial weight in the feature map after channel weight enhancement. It obtains the output feature map enhanced by channel and space bi-dimensionally. The TA mechanism structure is shown in Figure 4.

**Figure 5:**
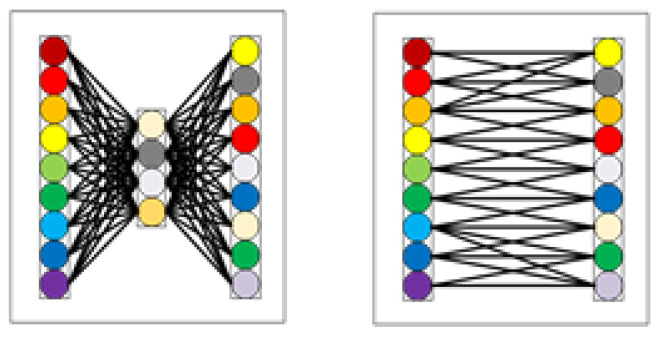
(**left**) MLP structure; (**right**) dynamically adjust channel; comparison diagram of MLP structure and dynamically adjusted k-nearest neighbor structure.

In the CAM construction process of the TA mechanism, the dynamically adjusted k-nearest neighbor structure in the ECA mechanism is used to replace the MLP structure in the CBAM to obtain local cross-channel feature information. The MLP structure requires all input nodes to participate in the calculation when generating each output node, which undoubtedly increases the computation of the attention mechanism. At the same time, the dimensionality reduction process in the MLP will reduce the dimension in the feature channel weight, which reduces the channel attention. In the dynamic adjustment of the k-nearest neighbor structure in ECA, when each output node is obtained, only K nodes related to the node are used for one-dimensional convolution calculation, which effectively reduces the module computation. At the same time, there is no dimension reduction process to overcome the problem of reducing channel attention. The MLP structure and the k-nearest neighbor structure of the dynamically adjusted channel are shown in Figure 5.

**Figure 6:**
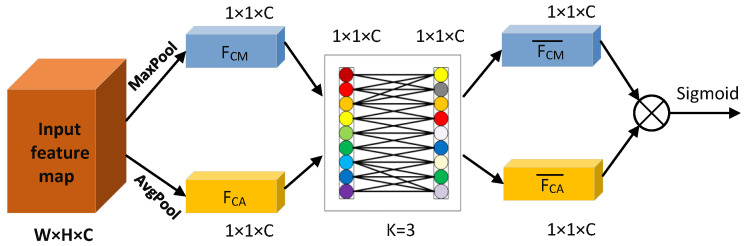
CAM construction process in TA when *k* = 3.

In Figure 5, *k* represents the number of adjacent input channels involved in predicting a certain output channel. The *k* can be adaptively determined according to the function of channels *C*, and the calculation method is shown in Formula (3):

(3) $$K=f\left(c\right)={\left|\begin{array}{c} \frac{\log_{2}C+b}{\gamma } \end{array}\right|}_{odd}.$$

where |•|${}_{odd}$ represents the nearest odd number to •, *b* = 1, and γ = 2. When constructing the attention weights of CAM, we use global average pooling (AvgPool) and maximum pooling (MaxPool) to obtain the channel attention weights *F_CA_* and *F_CM_*, respectively. The weight vectors *F_CA_* and *F_CM_* are then weight-learned using the adaptive k-nearest neighbor structure to obtain $\bar{F_{CA}}$ and $\bar{F_{CM}}$. We sum the $\bar{F_{CA}}$ and $\bar{F_{CM}}$ element by element to obtain a weight vector of 1 × 1 × *C*, which is the CAM weight block after activation by the Sigmoid function. When *k* = 3, the CAM construction process of TA is shown in Figure 6.

Given the input feature vector F, the calculation process of the CAM attention weight is shown in Formula (4):

(4) $$\begin{array}{cc} M_{c}\left(F\right) & =\sigma \left(C1D_{k}\left(\mathrm{AvgPool}\left(F\right)\right)+C1D_{k}\left(\mathrm{MaxPool}\left(F\right)\right)\right). \end{array}$$

In the formula, $M_{c}\left(F\right)$ refers to the calculated CAM weight block, σ(·) refers to the sigmoid activation function, $C1D_{k}\left(\cdot \right)$ represents the one-dimensional convolution process, and k refers to the used channels participating in the convolution number. In the SAM construction process in the TA mechanism, first we conduct global AvgPool and MaxPool operations on the input feature map in the spatial position to obtain two spatial attention weights $F_{S}A$ and $F_{S}M$, respectively, and combine $F_{S}A$ and $F_{S}M$ into a two-channel feature map through a channel concatenation operation. Then, the convolution layer composed of 7 × 7 convolution kernels is used to compress the channel of $F_{S}A$ and $F_{S}M$ to 1, and the size in the obtained feature map is W × H × 1. Finally, it is activated by the sigmoid function to obtain the SAM weight feature map.

Given the input feature vector F, the calculation process of the SAM weight is shown in Formula (5):

(5) $$M_{s}\left(F\right)=\sigma \left(f^{7\times 7}\left[\mathrm{AvgPool}\left(F\right);\mathrm{MaxPool}\left(F\right)\right]\right).$$

In the formula, $M_{s}\left(F\right)$ represents the calculated SAM weight block, σ(·) refers to the sigmoid activation function, and $f^{7\times 7}$ refers to the convolution process consisting of 7 × 7 size convolution kernels.

**Figure 7:**
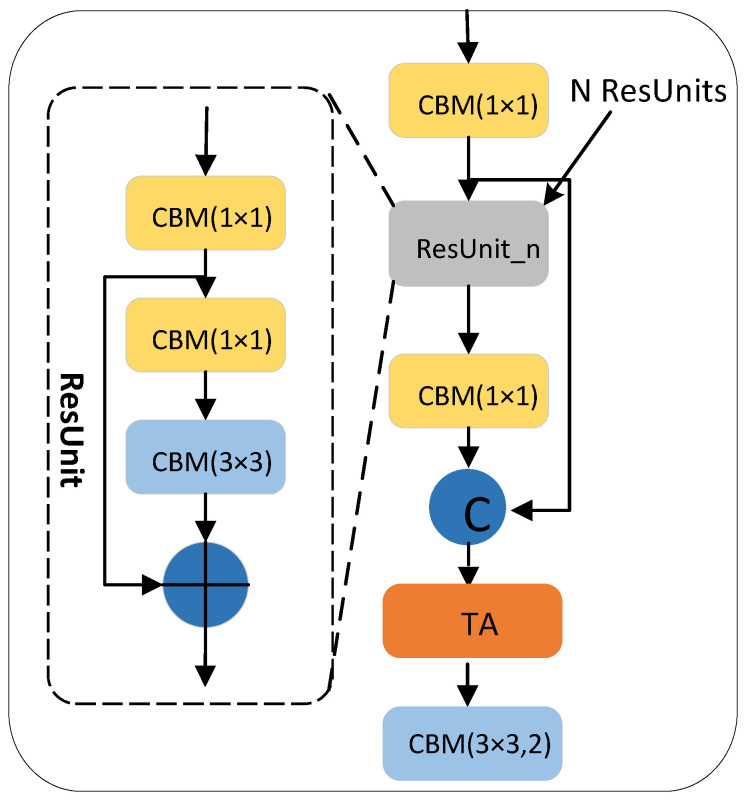
CSP with integrated TA modules.

In the CSPDarknet53 backbone network, a TA mechanism is added to each CSP structure to build a CSP structure that incorporates TA. In this structure, the TA mechanism is added after the residual unit operation, which enhances the weight of the feature layer from the two dimensions of space and channel with a small attention computation. The CSPDarknet53 backbone network with integrated TA mechanism attaches more attention weight to the pedestrian and enables the network to understand more feature information about pedestrian areas, further improving feature extraction capabilities throughout the algorithm. The improved CSP network is shown in Figure 7.

#### 3.2.3. PFM Pedestrian Area Feature Extraction Module

**Figure 8:**
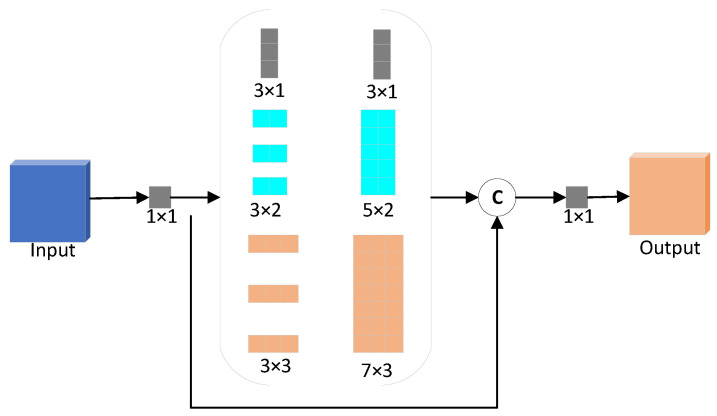
PFM structure diagram.

In the actual dense scene, the pedestrian target is different in the shooting position and angle. Its posture, body shape and height are also different, making the size distribution of pedestrian height and width uncertain. However, after clustering analysis of pedestrian annotation information in the dataset, it is found that dense pedestrian targets are often located in rectangular areas with specific aspect ratio prior information. Through different sized pooling operations, the SPP network in the original YOLOv4 performs multi-scale feature extraction on the feature layer in the network width, which verifies the rationality of extracting multi-scale features from the network width direction to a certain extent. However, the pooled receptive fields used by the SPP network are all square in size, which will cover more invalid background information outside the pedestrian area and interfere with the extraction in multi-scale features of dense pedestrians. For different scales extract pedestrian features, this paper still starts with a wider network and designs a Pedestrian-based Feature-extraction Module (PFM) based on the prior information of the aspect ratio on dense pedestrians. As shown in Figure 8, the PFM module has an area shape similar to the aspect ratio on dense pedestrians, which can effectively extract pedestrian features of different scales in the picture and better improve the algorithm’s extraction ability of multi-scale features about pedestrians.

As shown in Figure 8, the PFM module uses three branch convolutions with different convolutional kernel sizes to extract features, corresponding to the three sizes of 3 × 1, 3 × 2 and 3 × 3, respectively. To fit the aspect ratio on pedestrians and reduce memory consumption, the PFM module uses dilated convolutions [27] to extract pedestrian features. A larger pedestrian receptive field can be obtained with a small number of parameters, and the final receptive field size has three types: 3 × 1, 5 × 2 and 7 × 3. In the implementation process of the PFM module, the input feature layer channel is first compressed with the 1 × 1 convolution structure to reduce the model computation. Then, three different sizes of pedestrian sensory field convolution (3 × 1, 5 × 2 and 7 × 3) are used to obtain feature information. The resulting feature maps of different scales and the original feature maps are connected to the channel dimensions for identity mapping. Finally, the channel transformation is carried out by using 1 × 1 convolution to obtain the corresponding output feature map. The PFM module can broaden the network and extract pedestrian features of different sizes, thereby improving the detection accuracy of pedestrians.

### 3.3. Model Lightweight

In the YOLOv4-TP network, feature extraction obtains a three-scale feature map and then goes to the neck network for feature fusion. The original YOLOv4-TP network uses the PANet structure in the neck network, which has both deep-to-shallow semantic information fusion (up-sampling) and shallow-to-deep position information fusion (down-sampling). However, in the shallow-to-deep fusion process, the feature maps are mainly down-sampled, which is similar to the down-sampling process of the backbone network. Therefore, in the lightweight design process in the feature fusion stage, the down-sampling process of PANet is abandoned, and the ghost network fused with the TA mechanism is used to construct a one-way multi-scale feature fusion structure to achieve a lightweight feature fusion process.

#### 3.3.1. Ghost Module Incorporating TA

In the feature fusion process of the YOLOv4-TP network, many standard convolutions are used for channel transformation between feature layers of different scales, but standard convolution will cause a great amount of computation, which is not conducive to implementation in edge GPUs. Therefore, we use a ghost network that merges with the TA mechanism to build a new fusion structure. In a ghost network, convolution operations are divided into two processes. First, a standard convolution is used to compress the channels of the input feature map to obtain an intermediate feature map. It is then linearly transformed to generate more feature maps, connecting them together for the final output. The intermediate feature map is calculated by a standard convolution, and the process is shown in Formula (6):

(6) Y=X×K+b

where *X* is the feature map with *c* channels, *Y* is the generated n intermediate feature maps, *K* $\in R^{c\times k\times k\times n}$ is the standard convolution kernel used, *k* is the convolution kernel size, and *b* is the bias. To reduce the computation, a 1 × 1 pointwise convolution is used to generate intermediate feature maps. These intermediate feature maps are copied using a linear operation to generate m transformed feature maps for more output feature maps, and the process is as shown in Formula (7):

(7) $$Y_{ij}=\Phi_{i,j}\left(Y_{i}\right)\;\forall i=1,\ldots ,n,j=1,\ldots ,m.$$

In the formula, $Y_{i}$ represents the ith intermediate feature map, and $\Phi_{i,j}$ are the jth linear operation of the ith intermediate feature map to obtain the corresponding transformed feature map. Each $Y_{i}$ can obtain a maximum of m-1 transformed feature maps. The last time, no related linear transformation is performed, and the identity mapping in the intermediate feature map $Y_{i}$ is retained. Therefore, *n* × (*m* − 1) output feature maps can be generated for n intermediate feature maps.

**Figure 9:**
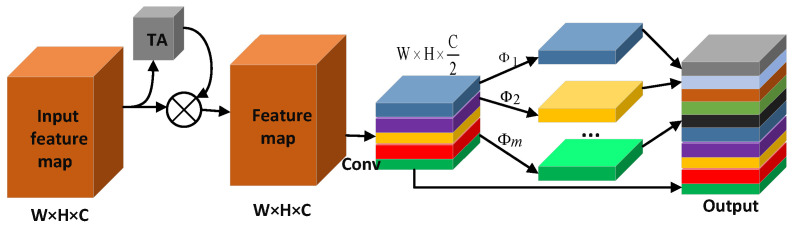
Ghost-TA module.

In the ghost module, the implementation of the linear operation is similar to the depth-wise convolution operation, which is to convolve a single feature map. Therefore, compared with standard convolution, linear operations require fewer parameters when obtaining more feature maps. Although the ghost module uses many linear operations to generate feature maps, the entire model has fewer parameters, but it is very effective. Therefore, the previously designed TA mechanism is added to the ghost module to build the ghost-TA module to enhance the attention to the visible area about pedestrians. Before the standard convolution of the input feature map, the pedestrian feature weight is enhanced by the TA mechanism to retain more original feature information. The standard convolution and subsequent linear operations are performed to generate the feature layer. Compared with the original ghost module, the ghost module after the integrated TA mechanism pays attention to the pedestrian feature information in the process of generating the feature map, which is conducive to improving the accuracy. The ghost-TA module is shown in Figure 9.

#### 3.3.2. One-Way Multi-Scale Feature Fusion Structure Design

**Figure 10:**
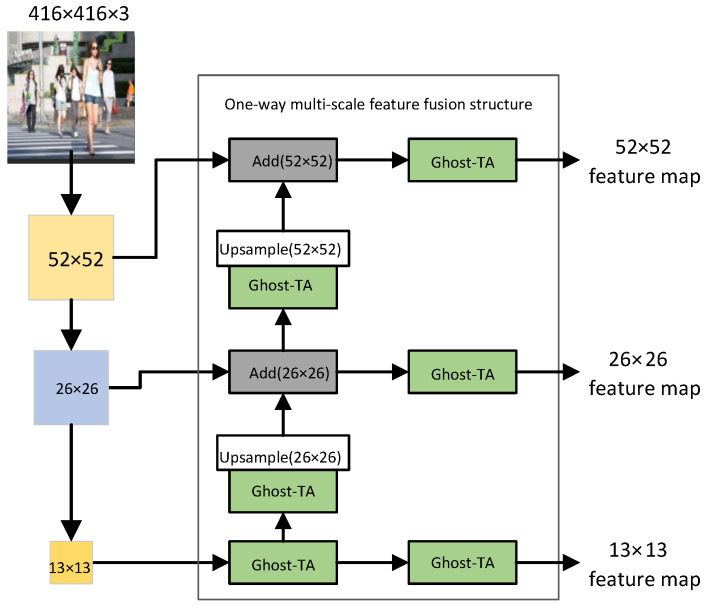
One-way multi-scale feature fusion structure.

The one-way multi-scale feature fusion structure abandons top-down sampling fusion and adopts a multi-scale feature fusion idea similar to the feature pyramid. It also realizes the lightweight of the fusion feature stage through the bottom-up up-sampling method. First, in this structure, the resolution in the feature map is increased by bilinear interpolation so that the two adjacent feature layers have the same size. Second, we use the ghost-TA module instead of the normal convolution and recombine the channels. Finally, the feature layers of different scales are fused by element-wise addition (Add). The one-way multi-scale feature fusion structure realizes the information interaction between different scale feature layers while reducing the computational complexity in the fusion process. When the image size is 416 × 416, the one-way multi-scale feature fusion structure is shown in Figure 10.

In Figure 10, Add represents that the fusion method between feature layers is an element-wise addition, (•) shows the size of the output feature layer after passing through this module, and ghost-TA is a lightweight module after replacing the standard convolution. The backbone network will down-sample the resolution of the input image by 8 times, 16 times and 32 times so that the size in the feature map will be changed, and the size of the changed feature map will be 52 × 52, 26 × 26 and 13 × 13. The one-way multi-scale feature fusion structure integrates the 13 × 13 feature map through the ghost-TA module to obtain two branches One branch obtains the 13 × 13 output feature map, and the other branch undergoes channel transformation through the ghost-TA module and then up-sampling so that it has the same dimensions as the input feature map. Finally, it adds the 26 × 26 input feature map element by element. After fusion, there are also two branches; one branch is used to obtain the fused 26 × 26 output feature map, and the other branch completes the fusion with the 52 × 52 input feature map in the same way. Finally, the last branch is used to obtain a 52 × 52 output feature map.

We have simplified the feature extraction process and feature fusion process of the YOLOv4-TP network, respectively. The lightweight YOLOv4-TP pedestrian detection algorithm is called the YOLOv4-TP-tiny, and the structure is shown in Figure 1.

## 4. Experiments

Since the improved model in this paper is suitable for edge GPUs, the computing power of the hardware equipment used in the experiment is not very high (GTX 960M).

- Dataset. We use an outdoor pedestrian detection benchmark dataset called WiderPerson [28] and a pedestrian dataset named CityPersons [29]. The widerperson contains five types of pedestrian entities: pedestrians, riders, partially-visible persons, ignore regions and crowd. We choose the first three pedestrian entities. The original dataset contains 13,382 images of which 4382 images are used as the test set without labels. The training set, validation set and test set required for the experiment are re-divided according to the ratio of 7:1:2, with 6300, 900 and 1800 images respectively. The citypersons has 2975, 500 and 1525 images for training, validation and testing datasets. Because the authors did not disclose test set annotation information, the experiment was conducted on 500 images of the verification testing set. The training set and the validation set are reclassified in a 9:1 ratio on 2975 training set pictures. Because this dataset is small, we only tested the effects of the YOLOv4-TP (not the lightweight version) and also compared the YOLOv4.
- Parameter settings. The initial learning rate is 1 × 10^−3^, and the batches are set to eight; the iteration period for training is set to 300 epochs. When the accuracy of the validation set does not increase after 10 epochs, the learning rate is attenuated by half.
- Evaluation metrics. Precision is used to evaluate the probability that the detection model predicts a positive class and is indeed a positive class. Recall is used to evaluate the ability of the detection model to predict all positive detection boxes. Only using precision and recall cannot evaluate the performance of the detection model well, so combining precision and recall can yield another comprehensive indicator for evaluating the detection model: the F1 value. The higher the F1 value is, the better the model performance. A single category of Average Precision (AP) is represented by an area surrounded by P–R curves and axes, and the performance of the model in detecting pedestrians can be evaluated. AP50 and AP75 refer to the AP value at an IoU threshold of 0.5 and 0.75, respectively. The AP value in the strict sense is obtained by averaging the APs under different IoU thresholds, that is, from 0.5 to 0.95, and calculating the APs for pedestrian detection under the IoU threshold with an interval of 0.05 and then taking the average value. In this experiment, the AP value in the strict sense is used as the average precision index.

In addition to the accuracy, the evaluation index of the pedestrian detection model is the detection speed. When evaluating the detection speed of pedestrian detection algorithms, frame per second (FPS) is a frequently used indicator. In the same hardware environment, the algorithm model can process the number of frames of pictures in one second.

### 4.1. Quantitative Evaluation

To conduct sufficient comparative experiments, the experiments in this section compare the YOLOv4-TP-tiny algorithm with lightweight detection algorithms such as SSD-Lite [30], the YOLOv3-Tiny [31], the YOLO-Slim and the YOLOv4-tiny algorithms on the widerperson dataset. It can be seen from Table 1 that the model parameter of the YOLOv4-TP-tiny algorithm is 22.5 MB, which is smaller than the four network models of SSD-Lite, the YOLOv3-tiny, the YOLO-Slim and the YOLOv4-tiny. In terms of precision, recall and AP, the YOLOv4-TP-tiny algorithm achieves 58.3%, 53.7% and 55.4%, respectively, which are higher than the other four models and have higher pedestrian detection accuracy. In terms of detection speed, the YOLOv4-TP-tiny algorithm reaches 31FPS, the YOLOv3-tiny and the YOLO-Slim, respectively, slightly higher than the YOLOv4-tiny, but the AP is improved by 3.8%. To sum up, compared with other lightweight detection models, the lightweight YOLOv4-TP-tiny algorithm model maintains a smaller model size and achieves higher detection accuracy.

As can be seen from Table 2, on the citypersons dataset the two-stage Faster R-CNN algorithm, although higher in average accuracy than the first-stage SSD algorithm, has a detection speed of only 28 FPS, which is lower than the SSD 19 FPS, showing that the one-stage algorithm has a great advantage in detection speed. Compared to the one-stage algorithm, the YOLOv4-TP algorithm, although slightly more than the other two algorithms in terms of model volume, has a clear advantage in average accuracy, reaching 72.9% compared to the YOLOv4, and the SSD increased by 4.5% and 13.1%, respectively. In terms of detection speed, the YOLOv4-TP algorithm reached 64FPS, which is 17FPS higher than the SSD, although slightly lower than the YOLOv4, but better in terms of accuracy, recall and average accuracy. Therefore, although the improved YOLOv4-TP model increases the model volume, its performance in detection accuracy is better than that of the other three network models, and it also achieves better detection speed.

### 4.2. Qualitative Evaluation

**Figure 11:**
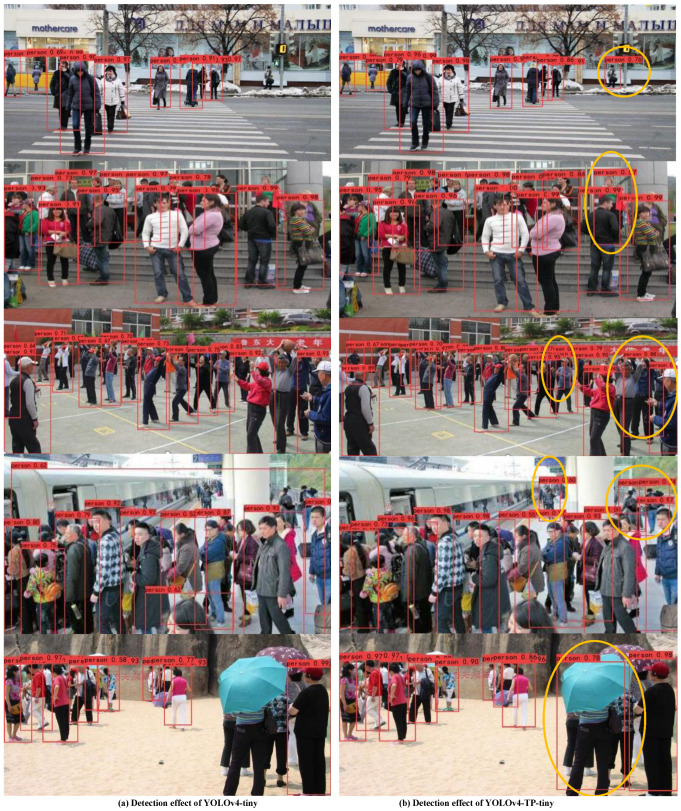
Comparison of detection effect between the YOLOv4-tiny and the YOLOv4-TP-tiny on the widerperson dataset.

The YOLOv4-TP-tiny algorithm draws on the lightweight idea of the YOLOv4-tiny algorithm. To clearly show the differences between the detection effects of the two algorithms, some pictures containing dense pedestrians are selected for detection. The detection effects comparing the YOLOv4 -tiny with the YOLOv4-TP-tiny are shown in Figure 11.

As can be seen from Figure 11a in Figure 11, the left column images are the detection effects of the YOLOv4-Tiny on the widerperson dataset. Figure 11b is the detection effect of the YOLOv4-TP-Tiny on the widerperson dataset we proposed. The yellow circle frame in the photo on the right is a pedestrian detected by the YOLov4-TP-Tiny, but the YOLOv4-Tiny did not did the pedestrian. In dense pedestrian scenes, the YOLOv4-TP-tiny is more accurate at locating target frames that obscure pedestrians than the YOLOv4-tiny is. At the same time, for pedestrians in different scales, the YOLOv4-TP-tiny algorithm cannot only recognize large-scale pedestrians closer, but also has a good recognition effect for small pedestrians farther away. However, the YOLOv4-tiny algorithm needs to be further improved in the case of more missed detection for small pedestrians. Therefore, compared with the YOLOv4-tiny algorithm, the YOLOv4-TP-tiny detection algorithm performs better in the detection of dense pedestrians.

### 4.3. Ablation Study

In this section, we conduct experiments in the test set of the widerperson dataset to verify the validity of each component in the YOLOv4-TP-Tiny. The components include TA and PFM; the results are shown in Table 3. Our proposed YOLOv4-TP improves AP from 61.3% to 64.9% compared to the YOLOv4; the results demonstrate that our TA module is more effective than the original architecture. The YOLOv4-TP-Tiny improves FPS from 29 to 31, which also improves AP from 51.6% to 55.4%. The results demonstrate that our PFM module is more effective than the original architecture. The YOLOv4-Mobilenet v2 and the YOLOv4-Ghostnet refer to the detection head of the YOLOv4, and the backbone networks are Mobiletv2 and Ghostnet, respectively. Because none of them are designed to detect pedestrians, many pedestrians may be missed in crowded environments, resulting in unsatisfactory results. It shows that our improved network is better at extracting pedestrian features and is more suitable for detecting pedestrians.

## 5. Conclusions and Feature Work

This paper proposes a pedestrian detection model called the YOLOv4-TP-Tiny based on the YOLOv4, which can better balance detection accuracy and speed on edge GPUs. We propose the TA module in the backbone network, which increases the attention of the network to the visible area of pedestrians. We also propose a new feature extraction method that can be better adapted to people of different sizes. Experiments have shown that the proposed module improves the effect of pedestrian detection, and the YOLOv4-TP-Tiny also balances detection accuracy and speed well.

However, the model we propose is still not the highest in terms of detection accuracy, and our follow-up work may be to further improve the accuracy of pedestrian detection. 

## Figures and Tables

**Table 1:** The comparison results between the YOLOv4-TP-tiny and other lightweight algorithms on the widerperson dataset.

Algorithm	Parameter/MB	Precision/%	Recall/%	AP/%	FPS
SSD-Lite	32.5	53.1	46.4	48.7	22
YOLOv3-tiny	35.6	51.6	43.7	46.9	20
YOLO-Slim	28.7	57.8	51.2	53.4	25
YOLOv4-tiny	25.4	55.9	50.5	51.6	29
YOLOv4-TP-tiny	22.5	58.3	53.7	55.4	31

**Table 2:** The comparison results between the YOLOv4-TP and other algorithms on citypersons dataset.

Algorithm	Parameter/MB	Precision/%	Recall/%	AP/%	FPS
Faster R-CNN	528.7	69.4	63.6	67.4	28
SSD	231.6	61.7	56.8	59.8	47
YOLOv4	244.2	70.9	67.2	68.4	69
YOLOv4-TP	251.3	73.7	69.4	72.9	64

**Table 3:** The performance of different components of our model on the test set of widerperson.

Model	FPS	Parameter/MB	AP/%
YOLOv4	8	244.2	61.3
YOLOv4-Tiny	29	25.4	51.6
YOLOv4-Mobilenetv2	21	46.8	48.3
YOLOv4-Ghostnet	25	42.7	50.7
YOLOv4-TP	7	251.3	64.9
YOLOv4-TP-Tiny	31	22.5	55.4

## Data Availability

The data used to support the finding of the current study are available from the corresponding author upon request. Prior studies (and datasets) are cited at relevant places within the text as reference [28].
